# Beliefs, barriers and hesitancy towards the COVID-19 vaccine among Bangladeshi residents: Findings from a cross-sectional study

**DOI:** 10.1371/journal.pone.0269944

**Published:** 2022-08-23

**Authors:** Md. Sharif Hossain, Md. Saiful Islam, Shahina Pardhan, Rajon Banik, Ayesha Ahmed, Md. Zohurul Islam, Md. Saif Mahabub, Md. Tajuddin Sikder

**Affiliations:** 1 Department of Public Health and Informatics, Jahangirnagar University, Savar, Dhaka Bangladesh; 2 Centre for Advanced Research Excellence in Public Health, Savar, Dhaka, Bangladesh; 3 Vision and Eye Research Institute, School of Medicine, Anglia Ruskin University, Young Street, Cambridge, United Kingdom; 4 Department of Unani, Government Unani and Ayurvedic Medical College & Hospital, Mirpur, Dhaka, Bangladesh; Qazvin University of Medical Sciences, ISLAMIC REPUBLIC OF IRAN

## Abstract

**Background:**

COVID‐19 vaccination acceptance is important, and combating hesitancy which is generally based on the individuals’ beliefs and perceptions is essential in the present pandemic. This study assesses COVID‐19 vaccine hesitancy and associated factors, beliefs and barriers associated with COVID-19 vaccination.

**Methods:**

A cross-sectional study was carried out among 492 Bangladeshi residents (76% male; mean age = 24.21 ± 4.91 years; age range = 18–50 years) prior to the nationwide mass COVID-19 vaccination campaign (September 28, 2021). A semi-structured e-questionnaire included three sections (demographic variables, beliefs around the vaccination, and perceived barriers regarding COVID-19 vaccination).

**Results:**

More than a quarter of participants (26.42%) were hesitant, 70.33% reported to accept the vaccine, and 3.25% refused to be vaccinated. While (54%) believed that mass vaccination would be the most effective method to combat the COVID-19 pandemic, concerns regarding the side effects of the vaccine (58%), inadequate vaccine trials before human administration (43%), commercial profiteering (42%), and mistrust of the benefits of the vaccine (20%) were also reported. In addition, other barriers including a short supply of vaccines, unknown future adverse effects (55%), low confidence in the health system (51%), doubts regarding its effectiveness (50%) and safety (45%), and insufficient information regarding potential adverse effects (44.7%) were reported. In bivariate analysis, variables such as current political affiliation, previous vaccination history, and health status were significantly associated with the COVID-19 vaccine uptake variable (acceptance, hesitancy, refusal). Regression analysis showed that participants who identified with the opposing current political parties, and not having been vaccinated since the age of 18 years were significantly more likely to report vaccine hesitancy.

**Conclusions:**

The current findings relating to COVID-19 vaccination demonstrate that government and policy makers need to take all necessary measures to ensure the effectiveness of the vaccination program among the Bangladeshi people.

## Introduction

The coronavirus disease-19 (COVID-19) caused by the severe acute respiratory syndrome coronavirus 2 (SARS-CoV-2) has become a significant threat worldwide [1, 2]. Globally, as of November 19, 2021, there have been over 255 million confirmed cases of COVID-19, including over 5 million deaths [3]. Bangladesh has been striving to control the impact of the COVID-19 pandemic since March 2020, despite insufficient health facilities [4]. Bangladesh was not well prepared to combat the spread of COVID-19 [5], and the COVID-19 testing mechanism for the nation has also received adverse reports [6]. Furthermore, COVID-19 has had one of the greatest health, economic, and communal impacts on lower- and middle-income countries (LMICs) like Bangladesh [7].

Vaccines are considered as the most important public health measure and most effective strategy to protect the population from the devastating outcomes of the COVID-19 pandemic [8, 9]. Over 300 COVID-19 vaccines are being developed, with 194 in non-clinical development and 130 in the clinical development phase [10]. Over 7.3 billion doses of coronavirus vaccines had been administered around the world as of November 18, 2021 [3]. Bangladesh is also moving forward with its vaccination program. On January 27, 2021, the Bangladesh government began the SARS-CoV-2 immunization campaign, announcing that interested people could register for vaccination through a dedicated website [11, 12]. In Bangladesh, as of November 19, 2021, a total of 52,995,353 (1^st^ doses; 30.67% population) and 33,999,865 (2^nd^ doses; 19.67% population) have been administered [13]. Although evidence on encouraging vaccination and its acceptance is useful, COVID-19 vaccine hesitancy has posed enormous challenges [14]. Doubts, mistrust, as well as the dissemination of misinformation increase vaccine hesitancy and resistance [15].

Vaccine hesitancy refers to a delay in acceptance or refusal of vaccination despite the availability of the vaccination service [16]. In 2019, the World Health Organization (WHO) declared vaccine hesitancy as one of the top ten global health threats [17]. In England, 55.8% of people who were surveyed reported a willingness to take the COVID-19 vaccine, while 34% were hesitant but leaning toward positive [18]. According to a study in the United States, willingness to be vaccinated decreased from 71% in April 2020 to 53.6% in October 2020. The majority of those who refused to get vaccinated were concerned about side effects and long-term health problems, as well as doubts about the vaccine’s efficiency [19]. Another study found that Taiwanese people had a poor willingness to obtain vaccinations among both healthcare workers (23.4%) and outpatients (30.7%) [20]. Furthermore, a systematic review and meta-analysis involving 19 studies from 11 countries revealed the pool rate of willingness to receive a COVID-19 vaccine among the general population was 60.1% [21]. Several risk factors associated with vaccine hesitancy and rejection have been reported in studies across different countries including socio-demographic factors (e.g., age, gender, marital status, employment status, income), cost, access to services, safety, and effectiveness [22–25]. Various factors including the perceived vaccine’s efficacy or safety, lobbying by anti-vaccination groups, and the accelerated vaccine research and production, were all found to be significant [26]. Evidence suggests that preventative behaviors or policies might be useful in pandemic control [27, 28]; nevertheless, it may not be always effective [29, 30]. These imply that it’s important to promote the COVID-19 vaccine and focus on herd immunity to prevent the COVID-19 pandemic.

A previous study has investigated COVID-19 vaccination reluctance in Bangladesh, reporting a 32.5% frequency in vaccine hesitancy [31], showing that Bangladesh, like the rest of the globe, is dealing with vaccine hesitancy. Moreover, perceived vaccine effectiveness and COVID-19 threat were also important predictors of COVID-19 vaccine hesitancy in Bangladesh [22]. To date, there are no previous studies in Bangladesh that have investigated beliefs, and barriers towards the COVID-19 vaccine. Thus, the present study was conducted to assess vaccine hesitancy and its associated factors, as well as to investigate the beliefs and barriers towards the COVID-19 vaccine in Bangladesh.

## Methodology

### Study participants and design

A cross-sectional anonymous survey was performed between April 30 to August 15, 2021. An online questionnaire collected self-reported data from the participants. The participants had to meet the following requirements in order to enroll in this study: ⅰ) being an adult (≥ 18 years old), ⅱ) social media user (Facebook, WhatsApp, etc.), ⅲ) currently living in Bangladesh, and ⅳ) having the willingness to complete the survey. Exclusion criteria of the present study were: ⅰ) being < 18 years old, ⅱ) incomplete survey, ⅲ) inconsistence response, and ⅳ) blank submitted response.

### Study procedure

The Google form online survey link was shared and marketed to the public through social media platforms such as Facebook, WhatsApp, Messenger, etc. At the beginning of the survey, formal informed consent was obtained where the aim of the study was clearly stated. Participants who agreed with all the terms and conditions were given access to the full questionnaire, otherwise, a blank survey form was submitted automatically. The questionnaire was adopted based on the previous studies [32–35]. It was pre-tested with small samples before starting the final data collection for acceptability and clarity.

### Sample size

The sample size was calculated using the following formula:

$$n=\frac{z^{2}\times p\times (1-p)}{d^{2}}$$

$$n=\frac{1.96^{2}\times 0.33\times (1-0.33)}{0.05^{2}}$$

339.75≈340

The critical value (z) included as 1.96 for a 95% confidence level. A prior study had reported vaccine hesitancy of 33% [31]. The precision limit or proportion of sampling error (d) of 5% was used and the sample size was calculated to be 340 people. As it was an online cross-sectional survey method, assuming a 40% non-response rate, a sample size of 475.65 ≈ 476 participants was estimated. This estimate was far exceeded by the current sample (492).

### Study instruments

The questionnaire consisted of three sections: (i) socio-demographic and other information; (ii) beliefs regarding COVID-19 vaccination; and (iii) barriers to COVID-19 vaccination.

#### Socio-demographic and vaccination acceptance

To record the socio-demographic information, participants were asked their age, sex, religion, marital status, education, occupation, family type (nuclear [two parents and their children]/ joint [family unit with more than two parents, extended family]) [36], monthly family income, and residence. Participants also answered questions regarding their current political affiliation, whether they practice any religion, tobacco smoking, chronic illness, previous vaccination history, self-perception of their own health status, the possibility of being infected with COVID-19 in the future, previous infection with COVID-19, or infection in a close social network, severity of infection if they have been infected with COVID-19, as well as self-rated knowledge about COVID-19 (Table 1). To assess vaccine hesitancy, participants were asked the following question (*“Would you take a COVID-19 vaccine if offered*?*”*) with three possible responses (definitely accept/ not sure [hesitant]/ definitely refuse) [32, 33].

**Table 1:** Distribution of COVID-19 vaccine acceptance, hesitancy and refusal among participants with examined variables.

Variables	Overall *N* = 492		Vaccine acceptance group		Vaccine hesitant group		Vaccine refusal group		*p*-value
	n	(%)	n	(%)	n	(%)	n	(%)	
**Age**									
Adults aged between 18–25 years	363	(73.8)	256	(70.5)	93	(25.6)	14	(3.9)	0.418*
Adults aged over 25 years	129	(26.2)	90	(69.8)	37	(28.7)	2	(1.6)	
**Sex**									
Male	373	(75.8)	265	(71.0)	96	(25.7)	12	(3.2)	0.817*
Female	119	(24.2)	81	(68.1)	34	(28.6)	4	(3.4)	
**Religion**									
Islam	433	(88.0)	304	(70.2)	115	(26.6)	14	(3.2)	0.484*
Hindu	48	(9.8)	36	(75.0)	11	(22.9)	1	(2.1)	
Buddha & Christian	11	(2.2)	6	(54.5)	4	(36.4)	1	(9.1)	
**Marital status**									
Single	421	(85.6)	298	(70.8)	108	(25.7)	15	(3.6)	0.384*
Married	57	(11.6)	41	(71.9)	15	(26.3)	1	(1.8)	
Separated/divorced/widowed	14	(2.8)	7	(50.0)	7	(50.0)	0	(.0)	
**Education**									
Higher secondary school or less	127	(25.8)	87	(68.5)	32	(25.2)	8	(6.3)	0.258*
Bachelor’s degree	276	(56.1)	199	(72.1)	71	(25.7)	6	(2.2)	
Master’s degree or higher	89	(18.1)	60	(67.4)	27	(30.3)	2	(2.2)	
**Occupation**									
Student	359	(73.0)	261	(72.7)	86	(24.0)	12	(3.3)	0.131*
Employee	56	(11.4)	39	(69.6)	15	(26.8)	2	(3.6)	
Businessman	25	(5.1)	17	(68.0)	6	(24.0)	2	(8.0)	
Unemployed	39	(7.9)	22	(56.4)	17	(43.6)	0	(.0)	
Others	13	(2.6)	7	(53.8)	6	(46.2)	0	(.0)	
**Family type**									
Nuclear	362	(73.6)	259	(71.5)	94	(26.0)	9	(2.5)	0.238
Joint	130	(26.4)	87	(66.9)	36	(27.7)	7	(5.4)	
**Monthly family income**									
<20,000 BDT	179	(36.4)	127	(70.9)	47	(26.3)	5	(2.8)	0.975
20,000–30,000 BDT	147	(29.9)	102	(69.4)	39	(26.5)	6	(4.1)	
>30,000 BDT	166	(33.7)	117	(70.5)	44	(26.5)	5	(3.0)	
**Residence**									
Urban	347	(70.5)	247	(71.2)	86	(24.8)	14	(4.0)	0.171*
Rural	145	(29.5)	99	(68.3)	44	(30.3)	2	(1.4)	
**Political affiliation**									
Ruling party	102	(20.7)	77	(75.5)	24	(23.5)	1	(1.0)	<0.001*
Opposing party	58	(11.8)	24	(41.4)	29	(50.0)	5	(8.6)	
Neutral	332	(67.5)	245	(73.8)	77	(23.2)	10	(3.0)	
**Obeying religious practices**									
Yes	386	(78.5)	273	(70.7)	100	(25.9)	13	(3.4)	0.908*
No	106	(21.5)	73	(68.9)	30	(28.3)	3	(2.8)	
**Tobacco use**									
Yes	97	(19.7)	67	(69.1)	26	(26.8)	4	(4.1)	0.773*
No	395	(80.3)	279	(70.6)	104	(26.3)	12	(3.0)	
**Chronic illness**									
Yes	75	(15.2)	53	(70.7)	20	(26.7)	2	(2.7)	1.000*
No	417	(84.8)	293	(70.3)	110	(26.4)	14	(3.4)	
**Family members suffering from any chronic illness**									
Yes	261	(53.0)	194	(74.3)	62	(23.8)	5	(1.9)	0.055
No	231	(47.0)	152	(65.8)	68	(29.4)	11	(4.8)	
**Any vaccination history after 18 years old**									
Yes	114	(23.2)	92	(80.7)	19	(16.7)	3	(2.6)	0.016*
No	378	(76.8)	254	(67.2)	111	(29.4)	13	(3.4)	
**Self-rated health status**									
Very poor	26	(5.3)	18	(69.2)	8	(30.8)	0	(.0)	0.002*
Poor	26	(5.3)	15	(57.7)	11	(42.3)	0	(.0)	
Moderate	177	(36.0)	119	(67.2)	46	(26.0)	12	(6.8)	
Good	210	(42.7)	164	(78.1)	44	(21.0)	2	(1.0)	
Very good	53	(10.8)	30	(56.6)	21	(39.6)	2	(3.8)	
**Self-rated COVID-19 knowledge level**									
Very poor	32	(6.5)	22	(68.8)	8	(25.0)	2	(6.3)	0.443*
Poor	15	(3.0)	8	(53.3)	6	(40.0)	1	(6.7)	
Moderate	231	(47.0)	158	(68.4)	66	(28.6)	7	(3.0)	
Good	185	(37.6)	138	(74.6)	41	(22.2)	6	(3.2)	
Very good	29	(5.9)	20	(69.0)	9	(31.0)	0	(.0)	
**Infected by COVID-19**									
Yes	41	(8.3)	31	(75.6)	8	(19.5)	2	(4.9)	0.358*
Not sure	180	(36.6)	124	(68.9)	53	(29.4)	3	(1.7)	
No	271	(55.1)	191	(70.5)	69	(25.5)	11	(4.1)	
**Close networks infected by COVID-19**									
Yes	162	(32.9)	116	(71.6)	40	(24.7)	6	(3.7)	0.597*
Not sure	118	(24.0)	77	(65.3)	38	(32.2)	3	(2.5)	
No	212	(43.1)	153	(72.2)	52	(24.5)	7	(3.3)	
**Possibility of COVID-19 infection**									
Low	163	(33.1)	113	(69.3)	42	(25.8)	8	(4.9)	0.351*
Medium	272	(55.3)	194	(71.3)	73	(26.8)	5	(1.8)	
High	57	(11.6)	39	(68.4)	15	(26.3)	3	(5.3)	
**Possibility of severity of COVID-19 infection**									
Low	122	(24.8)	85	(69.7)	34	(27.9)	3	(2.5)	0.818*
Medium	272	(55.3)	192	(70.6)	72	(26.5)	8	(2.9)	
High	98	(19.9)	69	(70.4)	24	(24.5)	5	(5.1)	

*Note*: BDT = Bangladeshi Taka; 1 BDT = 0.012 USD;

*Fisher’s exact test

#### Beliefs regarding COVID-19 vaccination

The second section included beliefs regarding COVID‐19 vaccination based on the previous literature [32, 35]. It included a total of 8 questions regarding beliefs towards COVID‐19 vaccination (e.g., *“I am worried about the side effects of vaccine*.*”*) with a three-point Likert scale (e.g., agree, undecided, disagree) (Table 3).

#### Barriers regarding COVID-19 vaccination

The third section included perceived hesitancy factors or barriers based on the previous literature [32, 34, 35]. It included a total of 10 questions regarding barriers towards COVID‐19 vaccination (e.g., *“Worry about unknown future effects of the vaccine*.*”*) with a three-point Likert scale (e.g., agree, undecided, disagree) (Table 4).

### Ethical consideration

The study protocol was approved by the Biosafety, Biosecurity and Ethical Committee, Jahangirnagar University, Savar, Dhaka, Bangladesh [BBEC, JU/ M 2021/COVID-19/10 (1)]. An informative statement was added at the beginning of the anonymous online questionnaire and the questionnaire included a consent which the participants had to agree to before they could take part in the study. Written e-consent was obtained from each participant. All responses were anonymous to ensure data confidentiality and participation in this study was completely voluntary.

### Statistical analysis

The Statistical Package for Social Sciences (SPSS; version 25.0 [IBM Corporation, Armonk, New York, USA]) was used to analyze the data. The socio-demographic and other examined characteristics of the participants were presented using descriptive statistics such as frequencies (n) and percentages (%). The chi-square test was used to assess the significance of the association among COVID-19 vaccine uptake groups (acceptance, hesitancy, refusal) and sociodemographic variables. Binary logistic regression analysis was used to determine the associated factors of vaccine hesitancy. A *p*-value < 0.05 was regarded as statistically significant.

## Results

### Socio-demographic and other characteristics

The present sample included 492 participants with a mean age of 24.21 years (SD = 4.91) with an age range from 18–50 years (**Table 1**). More than half of the participants were male (75.8%). The majority of the participants were students (73.0%), single (85.6%), had a bachelor’s degree (56.1%), belonged to a nuclear family (73.6%), had monthly family income < 20,000 BDT (36.4%), and resided in urban areas (70.5%) (Table 1).

**Fig 1:**
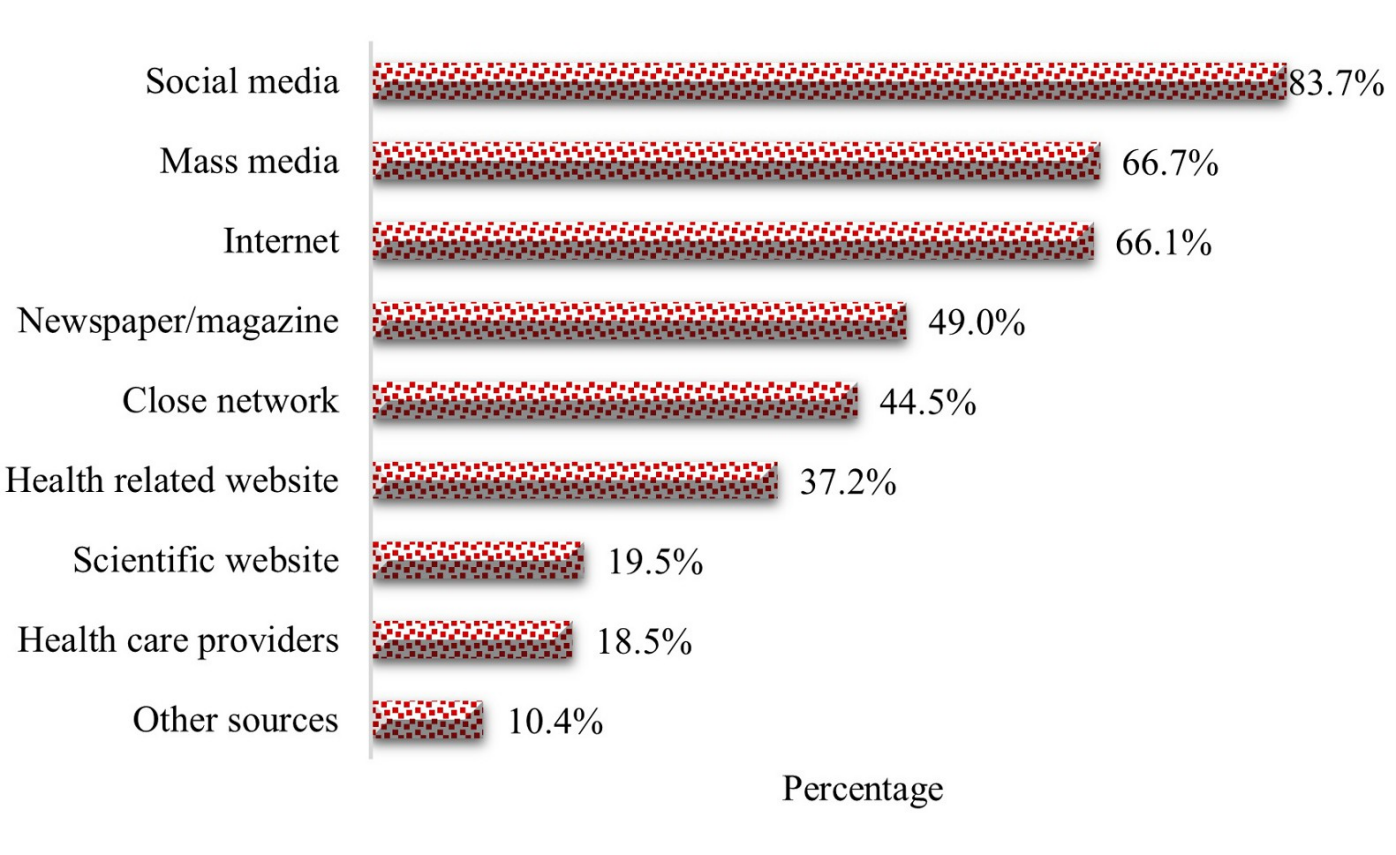
Information sources of COVID-19 and its vaccine.

A large proportion of participants reported neutral political affiliation (67.5%), being affiliated with a religious practice (78.5%), being non-smokers (80.3%), having no chronic illness (84.8%), having family members suffering from any chronic illness (53.0%), no history of vaccination after age 18 years (76.8%), and having a good self-rated health status (42.7%). About 83.7% of participants reported that social media (e.g., Facebook, Twitter, WhatsApp, etc.) was the main source of their information regarding COVID-19 and its vaccine, followed by 66.7% who reported other mass media (e.g., television, radio, etc.) and the internet (66.1%) (**Fig 1)**.

### COVID‐19 vaccine hesitancy and its associated factors

Out of the total 26.42% of respondents showed hesitancy to the COVID-19 vaccine, 70.33% were either already vaccinated or were willing to get vaccinated and 3.25% refused it definitely.

**Table 1** presents the profile of participants showing COVID-19 vaccine acceptance, hesitancy, and refusal among participants for socio-demographic and COVID-19 related variables. Bivariate analysis shows variables such as current political affiliation, previous vaccination history, and health status, were significantly (*p* < 0.05) associated with COVID-19 vaccine uptake groups (acceptance, hesitancy, and refusal).

In regression analysis, participants who reported who did not have a political affiliation to the current ruling party (they supported an opposition party) were 3.31 times more likely to have hesitancy towards the COVID-19 vaccine compared to those who reported neutral political affiliation (OR = 3.31; 95% CI = 1.87–5.88, *p* < 0.001). The participants who had been vaccinated for other diseases after the age of 18 years old were 0.48 times less likely to have hesitancy towards the COVID-19 vaccine compared to those who hadn’t been vaccinated after the age of 18 years (OR = 0.48; 95% CI = 0.28–0.83, *p* = 0.008) (Table 2).

**Table 2:** Factors of COVID‐19 vaccine hesitancy among participants by binary logistic regression analysis.

Variables	Hesitancy				OR (95% CI)	*p*-value
	*No*		*Yes*			
	n	(%)	n	(%)		
**Age**						
Adults aged between 18–25 years	270	(74.4)	93	(25.6)	0.856 (0.547–1.341)	0.498
Adults aged over 25 years	92	(71.3)	37	(28.7)	Ref.	
**Sex**						
Male	277	(74.3)	96	(25.7)	0.866 (0.547–1.373)	0.542
Female	85	(71.4)	34	(28.6)	Ref.	
**Religion**						
Islam	318	(73.4)	115	(26.6)	0.633 (0.182–2.202)	0.472
Hindu	37	(77.1)	11	(22.9)	0.520 (0.128–2.111)	0.361
Buddha & Christian	7	(63.6)	4	(36.4)	Ref.	
**Marital status**						
Single	313	(74.3)	108	(25.7)	0.345 (0.118–1.006)	0.051
Married	42	(73.7)	15	(26.3)	0.357 (0.107–1.188)	0.093
Separated/Divorced/Widowed	7	(50.0)	7	(50.0)	Ref.	
**Education**						
Higher secondary school or less	95	(74.8)	32	(25.2)	0.773 (0.423–1.415)	0.405
Bachelor’s degree	205	(74.3)	71	(25.7)	0.795 (0.470–1.346)	0.394
Master’s degree or higher	62	(69.7)	27	(30.3)	Ref.	
**Occupation**						
Student	273	(76.0)	86	(24.0)	0.368 (0.12–1.123)	0.079
Employee	41	(73.2)	15	(26.8)	0.427 (0.123–1.476)	0.179
Businessman	19	(76.0)	6	(24.0)	0.368 (0.089–1.532)	0.170
Unemployed	22	(56.4)	17	(43.6)	0.902 (0.256–3.181)	0.872
Others	7	(53.8)	6	(46.2)	Ref.	
**Family type**						
Nuclear	268	(74.0)	94	(26.0)	0.916 (0.584–1.437)	0.702
Joint	94	(72.3)	36	(27.7)	Ref.	
**Monthly family income**						
<20,000 BDT	132	(73.7)	47	(26.3)	0.987 (0.611–1.594)	0.958
20,000–30,000 BDT	108	(73.5)	39	(26.5)	1.001 (0.606–1.656)	0.996
>30,000 BDT	122	(73.5)	44	(26.5)	Ref.	
**Residence**						
Urban	261	(75.2)	86	(24.8)	0.756 (0.492–1.162)	0.203
Rural	101	(69.7)	44	(30.3)	Ref.	
**Political affiliation**						
Ruling party	78	(76.5)	24	(23.5)	1.019 (0.604–1.72)	0.944
Opposing party	29	(50.0)	29	(50.0)	3.312 (1.865–5.881)	**<0.001**
Neutral	255	(76.8)	77	(23.2)	Ref.	
**Obeying religious practices**						
Yes	286	(74.1)	100	(25.9)	0.886 (0.548–1.432)	0.620
No	76	(71.7)	30	(28.3)	Ref.	
**Tobacco use**						
Yes	71	(73.2)	26	(26.8)	1.025 (0.620–1.693)	0.924
No	291	(73.7)	104	(26.3)	Ref.	
**Chronic illness**						
Yes	55	(73.3)	20	(26.7)	1.015 (0.582–1.77)	0.959
No	307	(73.6)	110	(26.4)	Ref.	
**Family members suffering from any chronic illness**						
Yes	199	(76.2)	62	(23.8)	0.747 (0.500–1.116)	0.154
No	163	(70.6)	68	(29.4)	Ref.	
**Any vaccination history after 18 years old**						
Yes	95	(83.3)	19	(16.7)	0.481 (0.280–0.826)	**0.008**
No	267	(70.6)	111	(29.4)	Ref.	
**Self-rated health status**						
Poor	15	(57.7)	11	(42.3)	1.650 (0.528–5.158)	0.389
Moderate	131	(74.0)	46	(26.0)	0.790 (0.322–1.939)	0.607
Good	166	(79.0)	44	(21.0)	0.596 (0.243–1.462)	0.259
Very good	32	(60.4)	21	(39.6)	1.477 (0.544–4.007)	0.444
Very poor	18	(69.2)	8	(30.8)	Ref.	
**Self-rated COVID-19 knowledge level**						
Poor	9	(60.0)	6	(40.0)	2.000 (0.541–7.388)	0.298
Moderate	165	(71.4)	66	(28.6)	1.200 (0.513–2.806)	0.674
Good	144	(77.8)	41	(22.2)	0.854 (0.357–2.043)	0.723
Very good	20	(69.0)	9	(31.0)	1.350 (0.440–4.146)	0.600
Very poor	24	(75.0)	8	(25.0)	Ref.	
**Infected by COVID-19**						
Yes	33	(80.5)	8	(19.5)	0.710 (0.313–1.610)	0.412
Not sure	127	(70.6)	53	(29.4)	1.222 (0.802–1.862)	0.351
No	202	(74.5)	69	(25.5)	Ref.	
**Close networks infected by COVID-19**						
Yes	122	(75.3)	40	(24.7)	1.009 (0.628–1.622)	0.971
Not sure	80	(67.8)	38	(32.2)	1.462 (0.889–2.402)	0.134
No	160	(75.5)	52	(24.5)	Ref.	
**Possibility of COVID-19 infection**						
Low	121	(74.2)	42	(25.8)	0.972 (0.489–1.930)	0.935
Medium	199	(73.2)	73	(26.8)	1.027 (0.537–1.963)	0.935
High	42	(73.7)	15	(26.3)	Ref.	
**Possibility of severity of COVID-19 infection**						
Low	88	(72.1)	34	(27.9)	1.191 (0.649–2.186)	0.572
Medium	200	(73.5)	72	(26.5)	1.110 (0.651–1.892)	0.701
High	74	(75.5)	24	(24.5)	Ref.	

*Note*: BDT = Bangladeshi Taka; 1 BDT = 0.012 USD

### Self-rated COVID-19 knowledge level and vaccine groups

**Fig 2:**
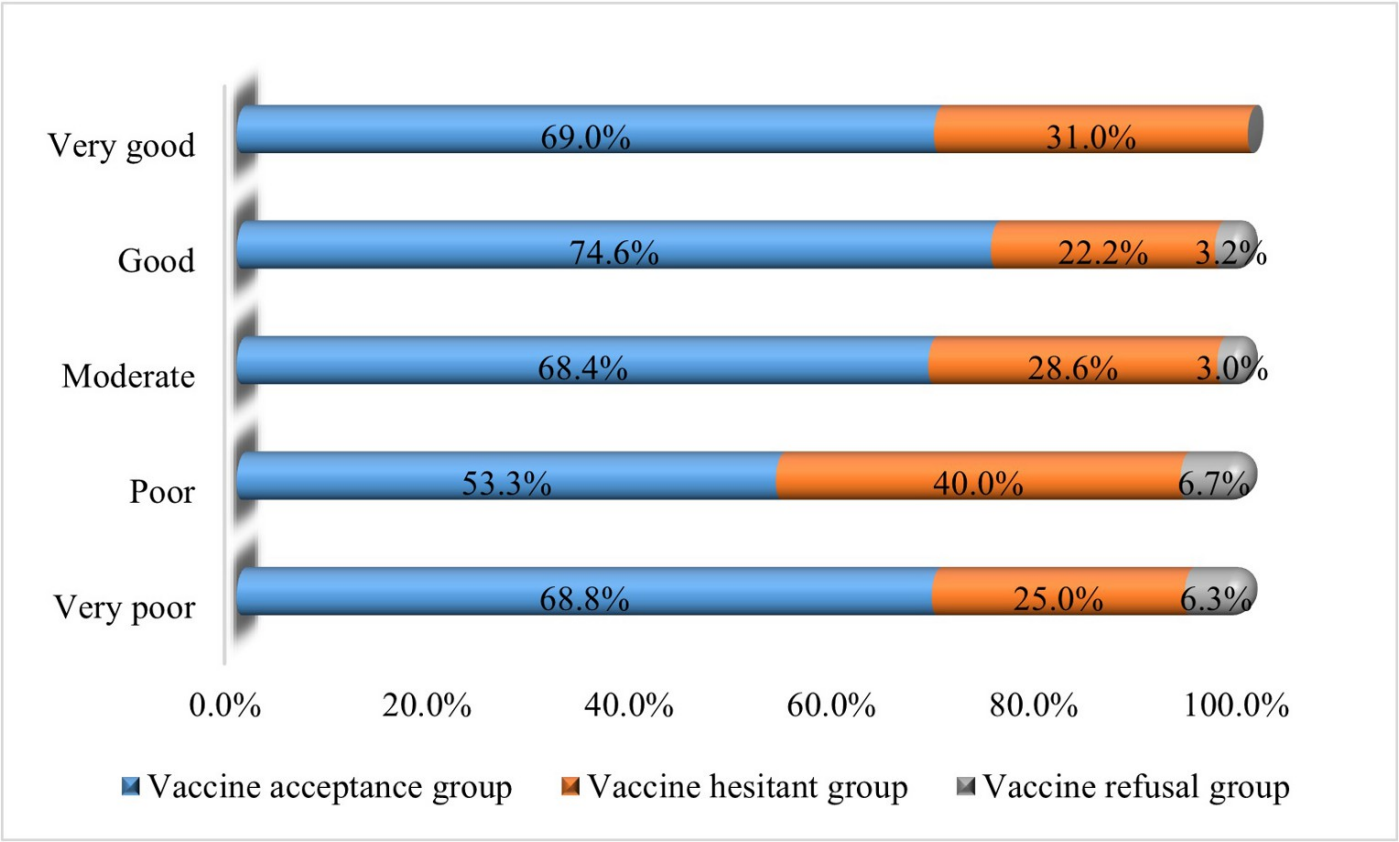
Self-rated COVID-19 knowledge level and vaccine groups.

Participants who reported having good knowledge regarding COVID-19 had a higher level of acceptance rate and a lower level of hesitancy towards the COVID-19 vaccine (Fig 2).

### Participants’ beliefs regarding COVID-19 vaccination

Only 53.9% of the participants believed that mass vaccination would be the most effective method to combat the COVID-19 pandemic. A high proportion had concerns regarding the side effects of the vaccine (57.9%). In addition, participants also reported to, they had concerns about whether the vaccines were not tested adequately (42.7%), commercial profiteering (42.3%), and mistrust of the benefits of the vaccine (20.1%). One-fifth (22.6%) had no prior bad experience with any vaccines or adverse reactions. A third of participants did not believe themselves to be at an elevated risk of contracting COVID‐19 (32.3%) and 39.4% did not perceive of being at a considerable risk of developing complications if they were infected (Table 3).

**Table 3:** Participants’ beliefs regarding COVID-19 vaccination.

Variables	n	(%)
**I am worried about the side effects of vaccine.**		
Undecided	99	(20.1)
Disagree	108	(22.0)
Agree	285	(57.9)
**I generally mistrust the benefits of vaccine.**		
Undecided	311	(63.2)
Disagree	82	(16.7)
Agree	99	(20.1)
**I believe that the way to overcome the COVID‐19 pandemic is mass vaccination.**		
Undecided	119	(24.2)
Disagree	108	(22.0)
Agree	265	(53.9)
**I think that the vaccine was not tested for enough time.**		
Undecided	148	(30.1)
Disagree	134	(27.2)
Agree	210	(42.7)
**I have a prior bad experience with any vaccines and their adverse reactions.**		
Undecided	243	(49.4)
Disagree	111	(22.6)
Agree	138	(28.0)
**I perceive myself not at elevated risk to acquire COVID‐19.**		
Undecided	224	(45.5)
Disagree	109	(22.2)
Agree	159	(32.3)
**I think that I am not at a considerable risk of developing complications if I have been infected with COVID‐19.**		
Undecided	180	(36.6)
Disagree	118	(24.0)
Agree	194	(39.4)
**I am concerned about commercial profiteering.**		
Undecided	152	(30.9)
Disagree	132	(26.8)
Agree	208	(42.3)

### Barriers of COVID‐19 vaccination among the study participants

The most‐reported barriers to COVID‐19 vaccination were limited vaccine supply and perceived that other people might be more in need of the vaccine (57.3%), concerns around the unknown future side-effects of the vaccine (55.3%), low confidence in the health system to handle a pandemic (51.2%), doubts about the vaccine effectiveness (50.2%) and safety (45.3%), and insufficient information regarding the potential adverse effects (44.7%). Other barriers to COVID-19 vaccination were insufficient trust in the manufacturers of vaccines (39.6%), the assumption that herd immunity would be protective without the vaccine (37%), insufficient information regarding the vaccine (35.4%), and the impact of pandemic being greatly exaggerated (31.9%) (Table 4).

**Table 4:** The barriers of COVID‐19 vaccination among the study participants.

Variables	*Undecided*		*Disagree*		*Agree*	
	n	(%)	n	(%)	n	(%)
Worry about unknown future effects of the vaccine.	117	(23.8)	103	(20.9)	272	(55.3)
Doubt in vaccine safety.	175	(35.6)	94	(19.1)	223	(45.3)
Doubt in vaccine effectiveness.	152	(30.9)	93	(18.9)	247	(50.2)
Vaccines are limited and other people need it more than me.	135	(27.4)	75	(15.2)	282	(57.3)
Insufficient trust in the vaccination source (producer).	177	(36.0)	120	(24.4)	195	(39.6)
Insufficient information regarding the vaccine.	207	(42.1)	111	(22.6)	174	(35.4)
Insufficient information regarding the potential adverse effects.	179	(36.4)	93	(18.9)	220	(44.7)
The impact of the coronavirus is being greatly exaggerated.	230	(46.7)	105	(21.3)	157	(31.9)
Low confidence in health system to handle pandemic.	151	(30.7)	89	(18.1)	252	(51.2)
Herd immunity will protect me even if I don’t have the vaccine.	167	(33.9)	143	(29.1)	182	(37.0)

## Discussion

Vaccine hesitancy is a key barrier in worldwide efforts to contain the current pandemic. Understanding and increasing acceptance of the COVID-19 vaccine is essential in developing an effective post-pandemic strategy [37]. In the present study, 26.42% of participants were hesitant, 70.33% were already vaccinated or were willing to get vaccinated, and 3.25% refused the vaccine. Various factors were significantly associated with COVID-19 vaccine uptake groups included political affiliation, vaccination history, and health status. In the regression analysis, participants who had not received other vaccines after the age of 18, and were politically opposing party were significantly associated with COVID-19 vaccine hesitancy.

A previous study conducted in Bangladesh reported that about one-third of the participants (32.5%) expressed vaccination hesitancy, which was slightly higher than in the current study (26.42%) [31]. A web-based anonymous cross-sectional study of the Bangladeshi general population found that 61.16% were willing to accept the COVID-19 vaccine, which was lower than the acceptance rate of 70.33% in the present study [38]. Another cross-sectional study of Bangladeshi adults by Parvej et al. [39], found that 67.04% of the total respondents were willing to receive a COVID-19 vaccine, which is close to the 70.33% reported in this study. Data from other parts of the world vary, for example, Saied et al. [32], in Egypt, reported that 35% of the participants were in the acceptance group, 46% were hesitant, and 19% refused among participants. The acceptance rate among the respondents of the current study is lower than shown in Australia (80%) [40], Denmark (80%) [41], India (79.3%-89.3%) [42, 43], and China, Korea, and Singapore (80%) [23], whilst being similar to studies reported from America (67%-69%) [44, 45], Saudi Arabia (64.7%) [46], Pakistan (70.25%) [47], Japan (65%) [48], and (after confirming the vaccine’s safety and efficacy) Russia (63.2%) [1].

This study found that political affiliation was significantly associated with COVID-19 vaccine acceptance, hesitancy and refusal. Individuals belonging to opposing parties had a significantly lower rate of COVID-19 vaccine acceptance (41.4%) compared to those identifying themselves as being neutral (73.8%) or identifying themselves with the current ruling party (75.5%). In the present study, participants who identified with the opposing party were 3.31 times more likely to report hesitancy than those who had no political affiliation. A comprehensive review and meta-analysis of vaccination hesitancy in LMICs found that vaccine hesitancy was associated with confidence in healthcare professionals, the health system, the government, and friends and family members [49]. The current study’s findings, however, are somewhat similar to a previous Bangladeshi study, which found statistically significant higher vaccine hesitancy among participants who identified themselves as politically affiliated to either the ruling or opposition party compared to those who did not have an affiliation [31].

In the present study, participants who had been vaccinated after the age of 18 were 0.48 times less likely to be hesitant about the COVID-19 vaccine than those who did not. The proportions of vaccine hesitancy and refusal were considerably lower among individuals who reported good health. Belief in vaccines to protect against infectious diseases and vaccines with minimal health risks were found to be important determinants in predicting willingness to receive the COVID-19 vaccine in Kuwait [50]. The varying information and confusion regarding new vaccinations and infections led to a reduced trust in the COVID-19 vaccination.

While the majority of participants believed that mass vaccination would be the most effective approach to combating the COVID-19 pandemic, there were concerns regarding the side effects of the vaccine, vaccine effectiveness and safety, and commercial profiteering. These findings go onto explain why, despite believing in the importance of the COVID-19 vaccine and agreeing that vaccination should be mandatory, participants are still hesitant due to a lack of certainty about vaccination safety and unknown potential adverse effects.

This study indicates that focusing on building trust in COVID-19 vaccines is important. This includes disseminating trusted information. In order to resolve this and clear misconceptions, communities should be included early on [51]. Since public trust in vaccination is fragile, the COVID-19 vaccination programs can only work if everyone believes the vaccinations are safe and effective [52]. Lucia et al. (2020) highlighted the need for transparency and responding to concerns regarding the efficiency and safety of vaccine development. It is critical to support COVID-19 vaccination through public statements and press releases, as well as to monitor and combat false news [53].

This study highlighted that the most common barriers to vaccination were short supply of vaccines, unknown future effects of the vaccine, low confidence in the health system, doubts about vaccine effectiveness and safety, and insufficient information regarding the potential adverse effects. Other barriers to COVID-19 vaccination were insufficient trust in the vaccination source (producer), believing that herd immunity will be enough protection in the absence of a vaccine, and the impression that the impact of coronavirus is greatly exaggerated. These results are similar to the prior studies which stated that concerns about the vaccine’s serious side effects, as well as a lack of trustworthy information, safety concerns, and vaccine mistrust lead to vaccine hesitancy [53, 54]. Misinformation about vaccines and a lack of advanced vaccination knowledge can cause hesitancy and lead to exaggeration of potential adverse effects [55]. It is expected that those hesitant would be more likely to accept vaccination if they were reassured and given reliable information. Gautam et al. (2020) investigated the respondents’ affordability and discovered that the majority of participants wanted a low-cost or free vaccine from the government [56]. Vaccine cost and efficacy appear to be important factors in vaccination acceptance [57].

Lessons learned from earlier outbreaks such as SARS, H1N1, and Ebola highlighted the crucial importance of health information in disease prevention and vaccine acceptance [58]. The World Health Organization has issued a warning that the world is facing a new type of disease known as an "infodemic," which rapidly spreads false news, misleading information, and misleading scientific claims [59]. Social media, conspiracy theories, and disinformation increase hesitancy substantially [60]. The most extensive sources of COVID-19 and vaccine information, according to research participants in the present study, were social media, mass media, and internet. The influence of social networks, such as family members, coworkers, and healthcare experts, on vaccination decision-making is significant [61]. According to Harapan et al., the majority of information regarding COVID-19 is spread through social or online media [25]. Perceptions are influenced by this misinformation [62]; leading to various conspiracy theories. As a result, accurate information on vaccination has been demonstrated to boost vaccine acceptance [63]. A large percentage of people regard health professionals as a credible source of COVID-19 information (75%) [44]. To improve trust and promote acceptance of the COVID-19 vaccine, it is important that healthcare expert groups interact with the public to convey accurate information around COVID-19 and the importance of vaccination [64].

Evidence indicated that the fear of COVID-19 could be an important factor contributing to vaccine hesitancy [65, 66]. However, this factor was not investigated in the present study. Trust could be a solution for vaccine acceptance improvement [67], and this echoes the present study finding that opposing the government party is a factor for vaccine refusal. Some important behavior theories (e.g., protection motivation theory and theory of planned behavior) are relevant to vaccine hesitancy and discussed in the literature [68–70]; however, none of these was investigated in the present study. The present study used a single-item question to assess the participants’ COVID-19 vaccine acceptance. A future study is warranted to get more information using the Drivers of COVID-19 Vaccination Acceptance Scale [71] as correct information may also be a key to improve COVID-19 vaccination [72].

### Limitations

There are some limitations of the current study that need to be considered when interpreting the results. Firstly, the selection bias was acknowledged as one of the key limitations due to the online survey, in which the majority of participants were younger adults (18–25 years), students, and males. As older people are more at risk of severe COVID-19, older people might be less hesitant towards a vaccine. Secondly, this study followed a cross-sectional study design that can not establish any causal inferences. Thirdly, the responses were based on self-reporting and may not be subject to self-reporting bias and a tendency to report socially desirable responses. Furthermore, the use of an online survey and convenience sampling may result in sampling bias as only participants who are digitally literate can take part. It would be useful to conduct this study in the future using other means such as face-to-face interviewing when it is safe to do so.

## Conclusions

This study found that over a quarter of respondents were hesitant to take the COVID-19 vaccine. Various beliefs and barriers regarding vaccination uptake were also explored among Bangladeshi residents. Participants who reported having good knowledge of COVID-19 also reported decreased vaccine hesitancy. Misinformation and misleading news about COVID-19 vaccines, particularly on social media platforms should be monitored. Accurate advice from healthcare experts and scientists would reduce uncertainty and build confidence around vaccination uptake and reduced mortality due to COVID-19.

## Supporting information

S1 File Data set. (XLSX)
